# Are VEGFR-TKIs effective or safe for patients with advanced non-small cell lung cancer?

**DOI:** 10.18632/oncotarget.4524

**Published:** 2015-06-19

**Authors:** Shuai Wang, Zhe Yang, Zhou Wang

**Affiliations:** ^1^ Department of Thoracic Surgery, Provincial Hospital Affiliated to Shandong University, Jinan, Shandong, People's Republic of China; ^2^ Department of Oncology, Provincial Hospital Affiliated to Shandong University, Jinan, Shandong, People's Republic of China

**Keywords:** lung cancer, angiogenesis inhibitors, VEGFR, TKIs, meta analysis

## Abstract

Vascular endothelial growth factor receptor tyrosine kinase inhibitors (VEGFR-TKIs) might be new therapeutic strategies for advanced non-small cell lung cancer (NSCLC). Here a total of 12,520 patients from 23 randomized controlled trials (RCTs) were enrolled to evaluate the efficacy and safety of VEGFR-TKIs quantitatively in advanced NSCLC. Compared with non-VEGFR-TKIs, VEGFR-TKIs regimen significantly improved progression-free survival (PFS) [hazard ratio (HR): 0.839, 95% confidence interval (CI): 0.805-0.874, *P* < 0.001], objective response rates (ORR) [relative risk (RR): 1.374, 95% CI: 1.193-1.583, *P* < 0.001] and disease control rates (DCR) (RR: 1.113, 95% CI: 1.027-1.206, *P* = 0.009), but not overall survival (OS) (HR: 0.960, 95% CI: 0.921-1.002, *P* = 0.060) for NSCLC patients. The RR of all-grade neutropenia, thrombocytopenia, hypertension, hemorrhage, fatigue, anorexia, stomatitis, diarrhea, rash, hand-foot skin reaction (HFSR) were increased in patients received VEGFR-TKIs. As for high-grade (≥ 3) adverse events (AEs), VEGFR-TKIs were associated with higher RR of neutropenia, thrombocytopenia, hypertension, fatigue, stomatitis, diarrhea, rash and HFSR. This study demonstrates VEGFR-TKIs improve PFS, ORR and DCR, but not OS in advanced NSCLC patients. VEGFR-TKIs induce more frequent and serious AEs compared with control therapies.

## INTRODUCTION

Standard therapy for advanced NSCLC is platinum-based palliative chemotherapy. Although 30-50% of NSCLC patients respond to platinum-based regimens, the median over survival (OS) is still 8-12 months [1, 2]. Traditional chemotherapy has limited clinical benefits. The “one size fits all” treatment model must be changed. The effective and less toxic drugs are urgently needed.

Over the past decade, progress of NSCLC biology promoted the development of targeted agents. Those targeted agents specifically inhibit vital signaling pathways, including epidermal growth factor receptor (EGFR) and vascular endothelial growth factor recepter (VEGFR) [3]. Currently, targeted therapies have emerged as novel therapeutic options and changed the treatment paradigm of NSCLC. VEGFR, a critical pathway in tumor progression, represents an important target in NSCLC [4]. There are two major categories of agents targeting VEGFR pathway: VEGF antibodies and VEGFR tyrosine kinase inhibitors (VEGFR-TKIs).

Several VEGFR-TKIs have been developed for targeted therapies in NSCLC, such as sorafenib, sunitinib, cediranib and vandetanib. The clinical efficacy of VEGFR-TKIs in advanced NSCLC, as a part of combination therapies or single agent had been evaluated [5-10]. But the results were inconsistent. A previous meta-analysis showed that chemotherapy plus VEGFR-TKIs significantly improved the progression-free survival (PFS), objective response rates (ORR) and disease control rates (DCR), but not overall survival (OS) [11]. Another meta-analysis suggested that VEGFR-TKIs significantly increased risk of death compared with non-VEGFR-TKIs [12]. In a recent meta-analysis, Shaodong H et al demonstrated that angiogenesis inhibitors had significant advantages over non-angiogenesis inhibitors in terms of PFS, OS, ORR and DCR in advanced NSCLC [13]. However, the meta analysis included VEGF antibody-based agents (bevacizumab, aflibercept and ramucirumab). Since then, several novel randomized controlled trials (RCTs) are emerging. Furthermore, previous studies showed different and even contradictory conclusions. The overall efficacy and safety of VEGFR-TKIs are undetermined. A more comprehensive review of previous studies is needed. In this study, we performed a pooled analysis of currently published RCTs to summarize the up-to-date evidence.

## RESULTS

### Literature search and study characteristics

**Figure 1:**
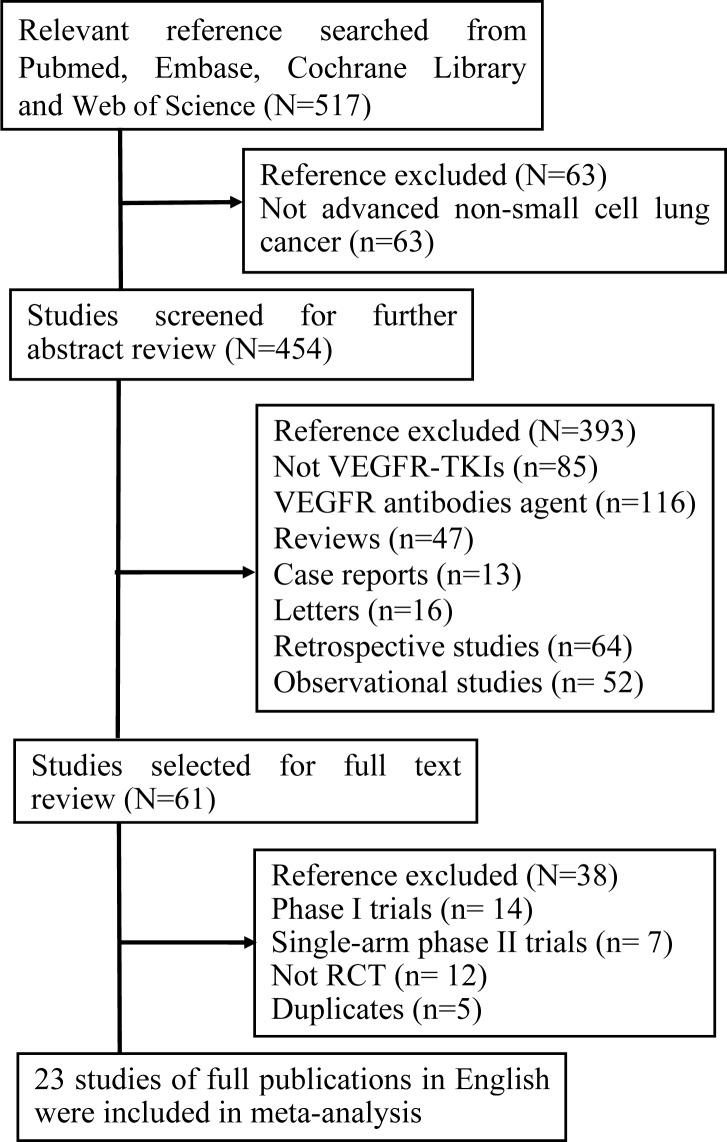
Study flow chart showing the process for selecting eligible publications N: the number of studies; RCT: randomized controlled trial.

A total of 517 articles were retrieved from initial electronic database and meeting abstracts. The selection steps in different phases were summarized in Figure 1. Finally, only 23 RCTs were included in our study. These RCTs enrolled a total of 12,520 patients (VEGFR-TKIs arm: 6487, control arm:6033). Among these 23 studies, 3 were on cediranib [14-16]; 1 on motesanib [10]; 2 on nintedanib [17, 18]; 1 on pazopanib [19]; 4 on sorafenib [9, 20-22]; 3 on sunitinib [23-25] and 9 on vandetanib [5-8, 26-30]. These RCTs comprised 12 phase II and 11 phase III clinical trials. Fourteen studies were published in recent three years. Eight trials were performed in first-line settings, 13 in ≥ second-line settings and 2 in maintenance. VEGFR-TKIs were compared with placebo in 6 trials and 17 trials were chemotherapy plus VEGFR-TKIs versus chemotherapy alone. The overall study quality was fair with a median Jadad score of 4, suggesting the quality of all RCTs was quite good. The characteristics of the 23 RCTs were listed in Table 1.

**Table 1:** Characteristics of studies included in the meta-analysis

Study	Year	Trial phase	Line	Arms	Cases	Stage IIIB/IV(n/n)	Median TT(months)	Medianage(years)	Objective response rate (n/N)	Disease control rate	Median PFS(months)	Median OS(months)	Jadad score
Laurie	2014	3	1	Cedi + PC	153	14/139	3.5	63	74/143	NA	5.5	12.2	4
[14]				Plac + PC	153	7/146	2.8	62	49/144		5.5	12.1	
Dy	2013	2	1	Cedi + GC	58	NA	2.5	65	11/58	NA	6.3	12	3
[15]				GC	29		2.8	64	6/29		4.5	9.9	
Goss	2010	2	1	Cedi + PC	126	NA	4.7	60	48/126	NA	5.6	NA	3
[16]				Plac + PC	125		4.4	58	20/125		5.0		
Scagliotti	2012b	3	1	Mote + PC	956	73/468	4.1	60	382/956	774/956	5.6	13	4
[10]				Plac + PC	949	79/470	4.1	60	247/949	702/949	5.4	11	
Reck	2014	3	2	Nint + Doc	655	148/399^1^	3.4	60	29/655	354/655	3.5	10.1	5
[17]				Plac + Doc	659	146/408	2.8	60	22/659	272/659	2.7	9.1	
Hanna	2013	3	2	Nint + Pem	353	NA	NA	60	NA	NA	4.4	12.2	4
[18]				Plac +Pem	360			59			3.6	12.7	
Scagliotti	2013	2	1	Pazo + Pem	62	2/59^2^	3.6	62	14/62	27/62	5.8	NA	2
[19]				DDP + Pem	35	3/32	2.7	64	12/35	26/35	5.3		
Wakelee	2012	2	>2	Sora	50	NA	2.0	64.5	NA	27/50	3.3	13.7	5
[20]				Plac	31		2.0	69		7/31	2	9
Paz-Ares	2012	3	1	Sora + GP	385	47/338	1.8	60	107/385	239/385	6	12.4	5
[21]				Plac + GP	387	47/340	2.0	58	100/340	244/340	5.5	12.5	
Spigel	2011	2	≥2	Sora + Erlo	111	NA	3.3	65	9/111	60/111	3.38	7.62	4
[22]				Plac + Erlo	55		3.3	65	6/111	21/111	1.94	7.23	
Scagliotti	2010	3	1	Sora + PC	464	44/420	3.9	62	127/464	232/464	4.6	10.7	4
[9]				Plac + PC	462	47/415	4.2	63	111/462	259/464	5.4	10.6	
Heist	2014	2	≥2	Suni + Pem	41	3/38	4.2	63	9/41	30/41	3.7	6.7	3
[23]				Pem	42	5/37	4.2	63	6/42	27/42	4.9	10.5	
Groen	2013	2	2 or	Suni + Erlo	65	1/63^3^	2.0	59	3/65	NA	2.8	8.2	5
[24]			3	Plac + Erlo	67	0/67	2.8	61	2/67		2.0	7.6	
Scagliotti	2012	3	≥2	Suni + Erlo	480	42/438	4.3	61	51/480	206/480	3.6	9.0	5
[25]	a			Plac + Erlo	480	32/448	4.4	61	33/480	168/480	2.0	8.5	
Aisner	2013	2	M	Vand	80	5/68^4^	2.8	63.5	15/80	NA	4.5	9.8	5
[26]				Plac	82	9/66	1.8	63	15/82		4.2	9.4	
Ahna	2013	2	M	Vand	75	15/60	2.0	61	14/75	NA	2.7	15.6	4
[27]				Plac	42	12/30	1.8	60.5	1/42		1.7	20.8	
Lee	2012	3	2 or	Vand	617	47/569	3.4	60	16/617	NA	1.9	8.5	3
[28]			3	Plac	307	17/289	2.5	60	2/307		1.8	7.8	
Natale	2011	3	2 or	Vand	623	106/51	2.1	61	75/623	254/623	2.6	6.9	3
[29]			3	Erlo	617	798/519	2.0	61	74/617	234/617	2.0	7.8	
de Boer	2011	3	≥2	Vand + Pem	256	37/219	3.4	60	49/256	146/256	4.1	10.5	4
[5]				Plac + Pem	278	46/232	2.8	60	22/278	128/278	2.8	9.2	
Herbst	2010	3	≥2	Vand + Doc	694	95/598^5^	2.8	59	120/694	413/694	4.0	10.6	5
[6]				Plac + Doc	697	106/590	3.0	59	71/697	380/697	3.2	10.0	
Natale	2009	2	≥2	Vand	83	14/69	NA	61	7/83	37/69	2.6	NA	3
[30]				Gefi	85	22/63		63	1/63	29/85	1.9		
Heymach	2008	2	1	Vand + PC	56	7/49	NA	60	18/56	NA	6.0	10.2	3
[7]				PC	52	5/47		59	13/52		5.8	12.6	
Heymach	2007	2	2	Vand + Doc	44	9/35	NA	60	8/44	28/44	4.0	7.9	3
[8]				Plac + Doc	41	13/28		58	5/41	23/41	2.8	13.4	

1: 105 patients with stage <IIIB in each arm and 3 patients missing the information; 2: 1 patient missing the information; 3:one subject in the sunitinib arm with no informed consent who was randomized in error; 4:7 patients with recurrence disease in each arm; 5: not recorded for one patient in each group; 6: Data not available for one patient in the vandetanib+ docetaxel arm. TT: treatment time; Cedi: cediranib; Plac: Placebo; Mote: motesanib; Nint: nintedanib; Pazo: pazopanib; Sora: sorafenib; Suni: sunitinib; Vand: vandetanib; GC: gemcitabine + carboplatin; DC: docetaxel + carboplatin; Doc: docetaxel; GP gemcitabine + cisplatin; Erlo: erlotinib; PC: paclitaxel + carboplatin; DDP:cisplatin; Pem: pemetrexed; Gefi: gefitinib; NA: not available; M: maintenance therapy; PFS: progression-free survival; OS: overall survival.

### Progression free survival

**Figure 2:**
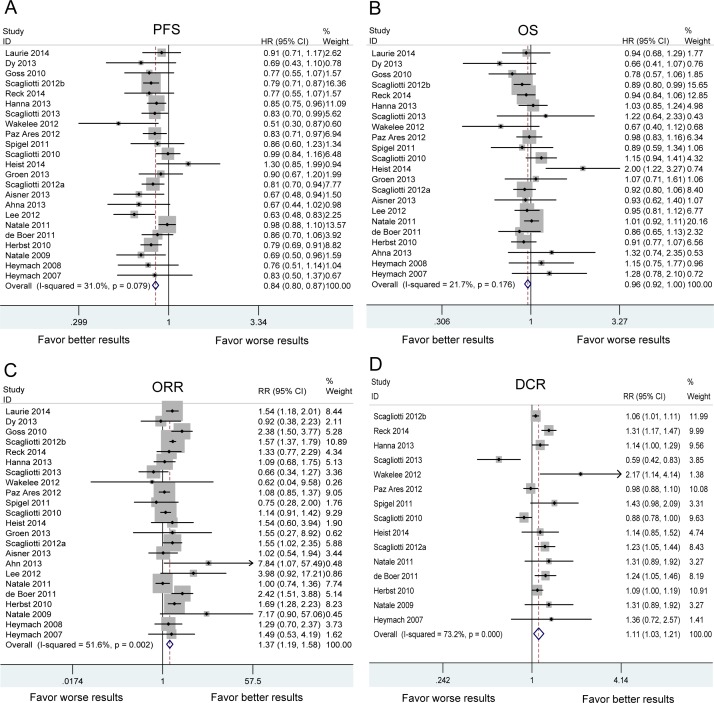
The pooled analysis of progression-free survival (PFS) A. overall survival (OS) B. objective response rate (ORR) C. and disease control rate (DCR) D. in NSCLC patients who received VEGFR-TKI therapies compared to control therapies HR: hazard ratio. RR: relative risk. Squares indicate study-specific HR or RR (size of the square reflects the study-specific statistical weight); horizontal lines indicate 95% confidence interval (CI); diamond indicates the summary HR or RR estimate with its 95% CI.

Twenty three studies reported the PFS of 6,487 patients in VEGFR-TKIs arm and 6,033 patients in control arm. Heterogeneity analysis revealed that there was no significant between-study heterogeneity (chi-squared = 31.88, d.f. = 22, *P* = 0.079, I-squared = 31.0%). A meta-analysis was therefore carried out using the fixed-effects model. A statistically significant improvement in PFS was observed favoring VEGFR-TKIs groups [hazard ratio (HR): 0.839, 95% confident intervals (CI): 0.805-0.874, *P* < 0.001) (Figure 2A).

**Figure 3:**
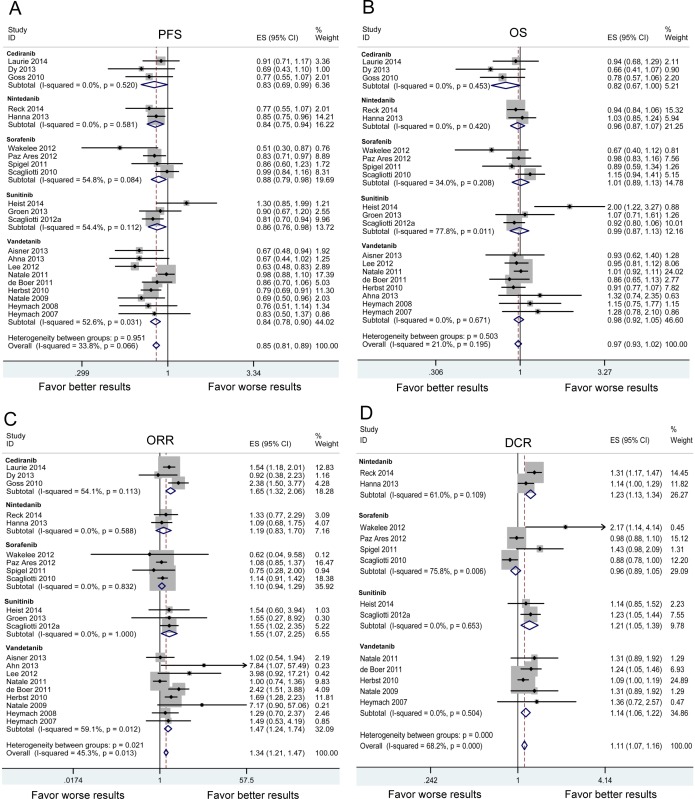
Subgroup analysis based on individual VEGFR-TKI in advanced NSCLC patients in terms of progressionfree survival (PFS) A. overall survival (OS) B. objective response rate (ORR) C. and disease control rate (DCR). D Squares indicate study-specific HR or RR (size of the square reflects the study-specific statistical weight); horizontal lines indicate 95% confidence interval (CI); diamond indicates the summary HR or RR estimate with its 95% CI.

**Figure 4:**
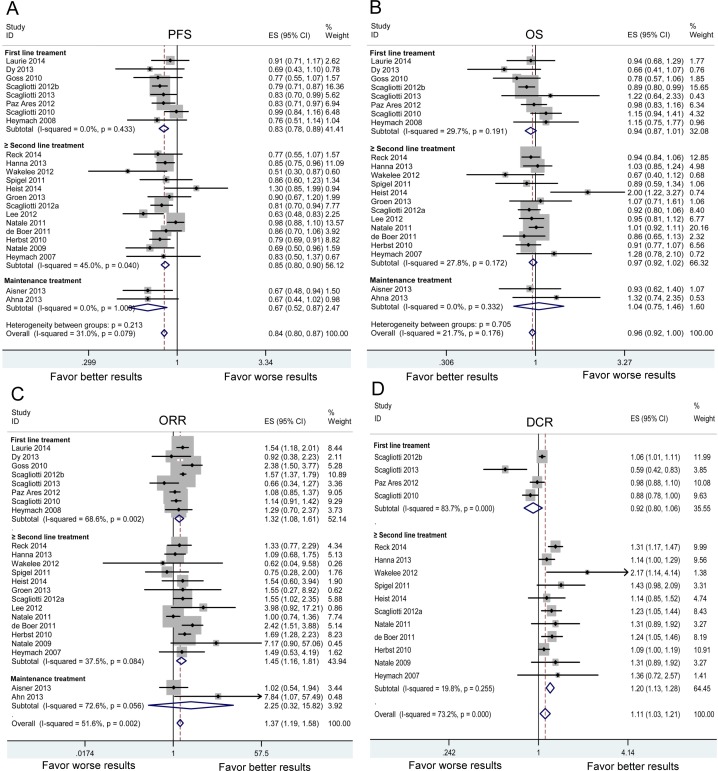
Subgroup analysis based on treatment line in advanced NSCLC patients in terms of progression-free survival (PFS) A. overall survival (OS) B. objective response rate (ORR) C. and disease control rate (DCR). D Squares indicate study-specific HR or RR (size of the square reflects the study-specific statistical weight); horizontal lines indicate 95% confidence interval (CI); diamond indicates the summary HR or RR estimate with its 95% CI.

**Figure 5:**
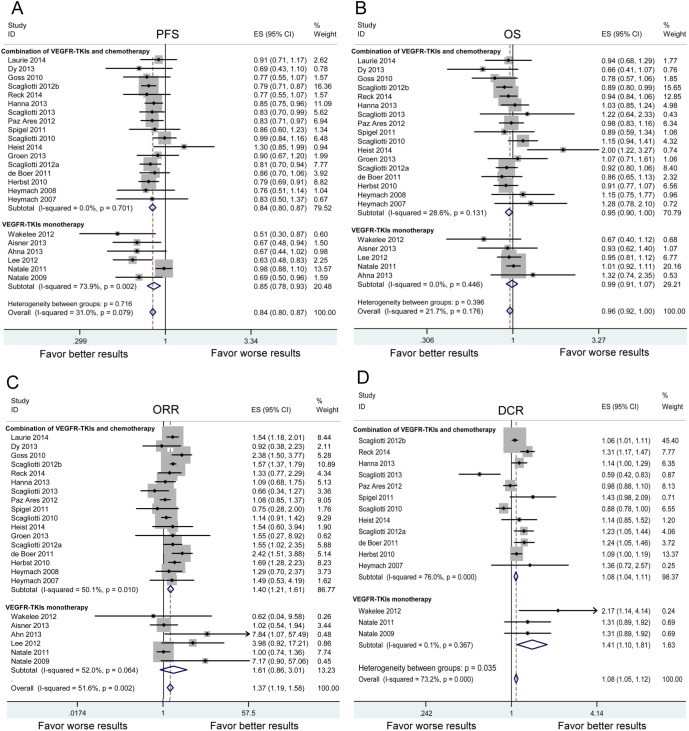
Subgroup analysis based on treatment regimen in advanced NSCLC patients in terms of progression-free survival (PFS) A. overall survival (OS) B. objective response rate (ORR) C. and disease control rate (DCR). D Squares indicate study-specific HR or RR (size of the square reflects the study-specific statistical weight); horizontal lines indicate 95% confidence interval (CI); diamond indicates the summary HR or RR estimate with its 95% CI.

Subgroups analyses were performed based on the individual VEGFR-TKI, treatment line and treatment regimen (Table 2). As shown in Figure 3A, significant PFS benefit was found in all VEGFR-TKIs. VEGFR-TKIs improved the PFS in first-line, ≥ second-line and maintenance treatment (Figure 4A). A statistically significant improvement in PFS was observed in both VEGFR-TKIs monotherapies (HR:0.707, 95%CI: 0.560-0.892) and combination therapies of VEGFR-TKIs with chemotherapy (HR:0.835, 95%CI: 0.798-0.875) (Figure 5A). We further performed meta-regression by the covariates including individual VEGFR-TKI, treatment line and treatment regimen. As was found in the subgroup analysis, individual VEGFR-TKI (*P* = 0.819), treatment line (*P* = 0.416) and treatment regimen (*P* = 0.261) did not result in the inter-study heterogeneity (Table 2).

**Table 2:** Results of subgroup analysis according to drug Class, treatment line and regimens for non-small cell lung cancer

Stratified analysis			No. of studies	Cases (TKIs /control arm)	Heterogeneity		Pooled HR/RR(95% CI)	Begg's Test		Egger's testP value	MetaregressionP value
					I^2^ (%)	P value		Z	P value		
**PFS**	TKIs	Cediranib	3	337/307	0.0	0.520	0.827 (0.687-0.994)	1.04	0.296	0.191	0.819
		Nintedanib	2	1008/1019	0.0	0.581	0.840 (0.748-0.943)	0.0	1.000	-	
		Sorafenib	4	1010/935	35.5	0.212	0.880 (0.792-0.977)	0.34	0.734	0.334	
		Sunitinib	3	586/589	54.4	0.112	0.861 (0.759-0.976)	1.04	0.296	0.231	
		Vandetanib	9	2528/2199	52.6	0.031	0.786 (0.697-0.887)	0.31	0.754	0.056	
	Line	1st	8	2260/2192	0.0	0.433	0.834 (0.783-0.889)	0.37	0.711	0.899	0.416
		≥2nd	13	4072/3717	45.0	0.040	0.831 (0.764-0.905)	0.33	0.743	0.244	
		Maintenance	2	155/124	0.0	1.000	0.670 (0.516-0.870)	0.00	1.000	-	
	Regimen	Combination	17	4959/4869	0.0	0.701	0.835 (0.798-0.875)	0.49	0.621	0.442	0.261
		Monotherapy	6	1528/1164	73.9	0.002	0.707 (0.560-0.892)	0.38	0.707	0.054
**OS**	TKIs	Cediranib	3	337/307	0.0	0.453	0.817 (0.668-1.000)	0.0	1.000	0.484	0.322
		Nintedanib	2	1008/1019	0.0	0.420	0.964 (0.873-1.066)	0.0	1.000	-	
		Sorafenib	4	1010/935	34.0	0.208	0.988 (0.838-1.166)	1.02	0.308	0.315	
		Sunitinib	3	586/589	77.8	0.011	1.193 (0.784-1.813)	1.04	0.296	0.348	
		Vandetanib	8	2445/2114	0.0	0.671	0.981 (0.917-1.050)	1.36	0.174	0.537	
	Line	1st	8	2260/2192	29.7	0.191	0.939 (0.872-1.012)	0.12	0.902	0.763	0.271
		≥2nd	12	3989/3632	27.8	0.172	0.969 (0.920-1.020)	−0.07	1.000	0.602	
		Maintenance	2	155/124	0.0	0.332	1.045 (0.749-1.457)	0.00	1.000	-	
	Regimen	Combination	17	4959/4869	28.6	0.131	0.949 (0.903-0.998)	1.61	0.108	0.158	0.227
		Monotherapy	5	1445/1079	0.0	0.446	0.988 (0.914-1.068)	−0.24	1.000	0.602	

Stratified analysis			No. of studies	Cases (TKIs /control arm)	Heterogeneity		Pooled HR/RR(95% CI)	Begg's Test		Egger's testP value	MetaregressionP value
					I^2^ (%)	P value		Z	P value		
**ORR**	TKIs	Cediranib	3	337/307	54.1	0.113	1.652 (1.101-2.479)	0.0	1.000	0.895	0.975
		Nintedanib	2	1008/1019	0.0	0.588	1.188 (0.832-1.696)	0.0	1.000	-	
		Sorafenib	4	1010/935	0.0	0.832	1.098 (0.936-1.287)	1.02	0.308	0.122	
		Sunitinib	3	586/589	0.0	1.000	1.548 (1.067-2.248)	0.0	1.000	0.659	
		Vandetanib	9	2528/2199	59.1	0.012	1.607 (1.155-2.234)	1.15	0.251	0.179	
	Line	1st	8	2260/2192	0.002	68.6	1.317 (1.077-1.611)	0.62	0.536	0.420	0.345
		≥2nd	13	4072/3717	37.5	0.084	1.427 (1.231-1.653)	0.06	0.951	0.576	
		Maintenance	2	155/124	72.6	0.056	2.254 (0.321-15.819)	0.00	1.000	-	
	Regimen	Combination	17	4959/4869	50.1	0.010	1.396 (1.212-1.608)	0.62	0.537	0.533	0.129
		Monotherapy	6	1528/1164	52.0	0.064	1.607 (1.056-3.014)	0.75	0.452	0.090
**DCR**	TKIs	Nintedanib	2	1008/1019	61.0	0.109	1.225 (1.069-1.404)	0.0	1.000	-	0.938
		Sorafenib	4	1010/935	75.8	0.006	1.081 (0.873-1.338)	1.02	0.308	0.095	
		Sunitinib	2	521/522	0.0	0.653	1.209 (1.052-1.389)	0.0	1.000	-	
		Vandetanib	5	1700/1718	0.0	0.504	1.137 (1.056-1.224)	0.0	1.000	0.065	
	Line	1st	4	1867/1833	83.7	0.000	0.920 (0.796-1.063)	1.70	0.089	0.068	0.023
		≥2nd	11	3390/3345	19.8	0.255	1.178 (1.121-1.238)	1.01	0.312	0.065
	Regimen	Combination	12	4501/4445	76.0	0.000	1.089 (1.004-1.182)	0.34	0.732	0.765	0.357
		Monotherapy	3	756/733	0.1	0.367	1.412 (1.101-1.812)	0.52	0.602	-

HR: hazard ratio, RR: relative risk, PFS: progression-free survival, OS: overall survival, ORR: objective response rate, DCR: disease control rate, TKIs: tyrosine kinase inhibitors. HR for PFS and OS. RR for ORR and DCR.

### Overall survival

The meta-analysis of OS was based on 22 RCTs provided the required data. Between-study heterogeneity could be ignored (chi-squared = 26.83, d.f. = 21, *P* = 0.176, I-squared = 21.7%). There was no significant difference between VEGFR-TKIs group and control group for OS (HR:0.960, 95%CI: 0.921-1.002, *P* = 0.060) (Figure 2B). In stratified analyses by individual VEGFR-TKI, significant OS benefit was not found in cediranib, nintedanib, sorafenib, sunitinib and vandetanib (Figure 3B). A positive effect of VEGFR-TKIs for OS was not observed in first-line treatment, ≥ second-line treatment, and maintenance treatment (Figure 4B). A statistically significant improvement in OS was observed in combination therapies of VEGFR-TKIs with chemotherapy, not in VEGFR-TKIs monotherapies (Figure 5B). Meta regression suggested that individual VEGFR-TKI (*P* = 0.322), treatment line (*P* = 0.271) and treatment regimen (*P* = 0.227) did not alter the pooled HR significantly (Table 2).

### Overall response rate and disease control rate

Twenty three RCTs provided information in detail about ORR, while DCR were suggested in only fifteen trials. The results of pooled analysis showed VEGFR-TKIs significantly improved ORR [relative risk (RR): 1.374, 95%CI: 1.193-1.583, *P* < 0.001] and DCR (RR: 1.113, 95%CI: 1.027-1.206, *P* = 0.009) (Figure 2C, 2D).

In stratified analyses regarding individual VEGFR-TKI, three VEGFR-TKIs (cediranib, sunitinib and vandetanib) resulted in a significant improvement of ORR (Figure 3C). Three agents (nintedanib, sunitinib and vandetanib) resulted in a significant increase of DCR (Figure 3D). The significant ORR benefit was found both in first-line and ≥ second-line treatment. However, better DCR was only found in ≥ second-line treatment (Figure 4D). Subgroup analysis showed that both monotherapy and combination therapy improved ORR and DCR (Figure 5C, 5D). Meta regression indicated that none of the examined factors were responsible for between-study heterogeneity on ORR, including individual VEGFR-TKI (*P* = 0.975), treatment line (*P* = 0.345) and treatment regimen (*P* = 0.129). In addition, individual VEGFR-TKI (*P* = 0.938) and treatment regimen (*P* = 0.357) did not result in significantly heterogeneity across studies on DCR. While, treatment line (*P* = 0.023) could be a important factor responsible for between-study heterogeneity on DCR (Table 2).

### Common adverse events

The common AEs were summarized in Table 3. The pooled analyses showed that the risks of all-grade neutropenia, thrombocytopenia, hypertension, hemorrhage, fatigue, anorexia, stomatitis, diarrhea, rash, HFSR were higher in patients receiving VEGFR-TKIs. The pooled RR indicated the risks of all-grade thromboembolism, dyspnea and neuropathy were comparable between VEGFR-TKIs and control group. However, the risk of all-grade anemia was decreased in patients treated with VEGFR-TKIs than those in control group (RR:0.820, 95%CI:0.683-0.984).

**Table 3:** Relative risk (RR) of common adverse events in advanced non-small cell lung cancer patients treated angiogenesis inhibitors

Adverse events	All grades			Grade ≥ 3		
	No. of studies	RR (95% CI)	P value	No. of studies	RR (95% CI)	P value
Anemia	9	0.820 (0.683-0.984)	0.033	12	0.821 (0.634-1.063)	0.135
Neutropenia	11	1.445 (1.091-1.914)	0.010	11	1.370 (1.065-1.762)	0.014
Thrombocytopenia	9	1.783 (1.170-2.716)	0.007	10	2.376 (1.710-3.302)	<0.001
Hypertension	12	4.012 (2.951-5.455)	<0.001	13	5.693 (3.668-8.834)	<0.001
Hemorrhage	6	1.968 (1.556-2.489)	<0.001	7	1.932 (0.933-3.997)	0.076
Thromboembolism	3	1.074 (0.644-1.792)	0.784	6	0.920 (0.620-1.366)	0.681
Fatigue	17	1.094 (1.002-1.195)	0.046	19	1.558 (1.334-1.820)	<0.001
Anorexia	10	1.209 (1.030-1.420)	0.020	12	1.327 (0.976-1.803)	0.071
Stomatitis	9	1.896 (1.377-2.611)	<0.001	8	3.835 (1.721-8.546)	0.001
Diarrhea	17	1.946 (1.655-2.287)	<0.001	20	2.747 (1.908-3.954)	<0.001
Dyspnea	10	0.997 (0.907-1.096)	0.870	13	0.873 (0.729-1.045)	0.139
Rash	17	1.661 (1.278-2.159)	<0.001	16	3.065 (1.626-5.778)	0.001
HFSR	5	3.909 (1.910-8.001)	<0.001	5	18.137 (6.657-49.414)	<0.001
Neuropathy	6	0.943 (0.823-1.082)	0.405	6	1.129 (0.704-1.808)	0.615

HFSR, hand-foot skin reaction; Hemorrhage: nose, CNS, lung and abdominal hemorrhage

To clarified the severity of AEs, we further analyzed the ≥ 3 grade AEs in VEGFR-TKIs and control group. Compared with the control group, the VEGFR-TKIs group showed a higher incidence of ≥ 3 grade neutropenia, thrombocytopenia, hypertension, fatigue, stomatitis, diarrhea, rash and HFSR. Patients receiving VEGFR-TKIs experienced a comparable risk of ≥ 3 grade anemia, hemorrhage, thromboembolism, anorexia, dyspnea and neuropathy (Table 3).

### Sensitivity analyses

**Figure 6:**
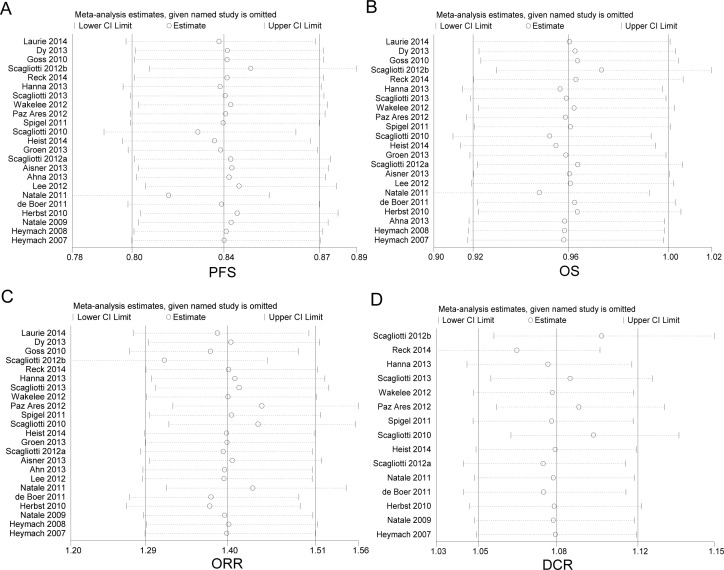
Sensitivity analysis of enrolled studies on progression-free survival (PFS) A. overall survival (OS) B. objective response rate (ORR) C. and disease control rate (DCR) D

We carried out sensitivity analyses to assess the stability of the results by sequentially omitting each study. The leave-one-out sensitivity analyses indicated that no individual study changed the pooled data qualitatively, suggesting that our results were stable and reliable (Figure 6).

### Publication bias

**Figure 7:**
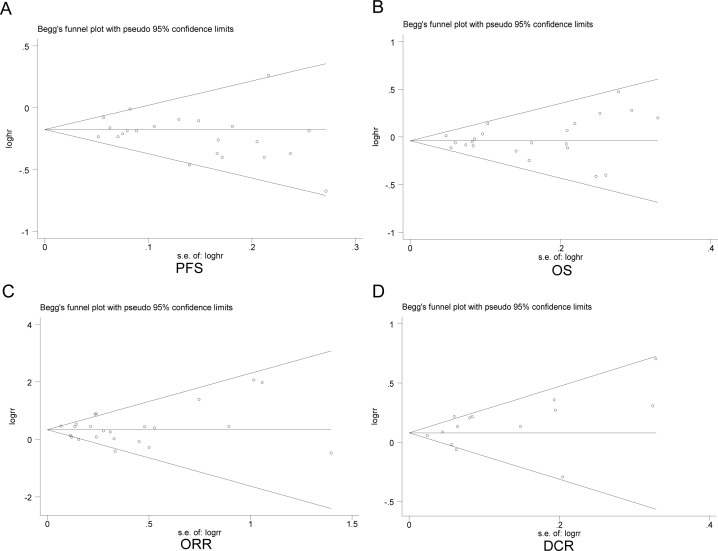
Begg's funnel plots of included studies on progression-free survival (PFS) **A.** overall survival (OS) **B.** objective response rate (ORR) **C.** and disease control rate (DCR) **D.**

The Begg's funnel plot and Egger's test were conducted to assess the publication bias. The shapes of the funnel plots seemed symmetrical in all meta-analyses, suggesting the absence of publication bias (Figure 7). Z-value (continuity corrected) of Begg's test in the pooled analysis on PFS was 0.98 (*P* = 0.328), 1.02 on OS (*P* = 0.310), 0.42 on ORR (*P* = 0.673) and 1.04 on DCR (*P* = 0.299). Egger's test showed that the t value (bias) of the meta-analyses on PFS was −1.54 (*P* = 0.138), 0.79 OS (*P* = 0.439), 0.12 on ORR (*P* = 0.439) and 0.99 on DCR (*P* = 0.338). As shown in Table 2, the results of Begg's test and Egger's test indicated no significant evidence for publication bias of the subgroup analyses. Bias from publications might not have a significant influence on the results of current meta-analyses. Therefore, we did not perform non-parametric “trim-and-fill” method to adjust pooled HRs or RRs.

## DISCUSSION

The present report with updated data improved our understanding about the efficacy and safety of VEGFR-TKIs in advanced NSCLC. The pooled results showed that VEGFR-TKIs were associated with significant improvement in PFS, ORR and DCR compared with control therapies. The basis of VEGFR-TKIs therapy is stemmed from recognition of the fact that VEGF is the most important growth factor for angiogenesis [4, 31]. VEGFR-TKIs compete with ATP for the activation domain and block intracellular VEGF signaling pathway [31]. Thus, VEGFR-TKIs result in regression of blood vessels, suppression of tumor angiogenesis and shrinkage of tumor volume.

Our results demonstrated significant association between improvement of PFS and VEGFR-TKIs (HR:0.839, 95%CI: 0.805-0.874, *P* < 0.001). However, the improvement of PFS with VEGFR-TKIs failed to translate into OS benefit (Figure 2). Our results were consistent with previous studies (11,13,20-36). One possible explanation is that PFS is a direct indicator for treatment efficacy, while the OS may be influenced by the post-progression treatment. Patients in VEGFR-TKIs group have longer PFS than control group. The improvement of symptom relief results in the possibility that patients could live with fewer symptoms for a longer time. Patients in control groups would receive more post-progression interventions. Therefore, they get important palliative benefit compared with patients in VEGFR-TKIs groups [11]. Another possible explanation is that tumor upregulates the expression of alternative pro-angiogenic factors, such as fibroblast growth factor, ephrin and angiopoietin after VEGFR-TKIs treatment [32]. Those factors compensate quickly the inhibition of VEGFR signal pathway. The exact reasons remain unclear. Thus, further studies with functional analyses are needed to address this issue.

In the present meta-analysis, we found the spectrum of VEGFR-TKIs associated AEs was consistent with previous studies [11, 13, 33]. Hypertension is a well-known AE of VEGFR-TKIs. VEGFR-TKIs induce vasoconstriction by inhibition flow-mediated dilation and nitroglycerin-mediated dilation [34]. Interestingly, occurrence of treatment-related hypertension is associated with benefit of VEGFR-TKIs [35]. However, it is unclear whether hypertension might exploited as an indicator for better PFS and OS in patients treated with VEGFR-TKIs. VEGF signal pathway plays a vital role in hematopoiesis. So, VEGFR-TKIs may lead to neutropenia and thrombocytopenia. However, the novel thing is that the risk of all-grade anemia was decreased in patients treated with VEGFR-TKIs (RR:0.820, 95%CI:0.683-0.984). One possible reason is the potential benefit of VEGFR-TKIs for reduction tumor burden. Another reason might be related to the increase of erythropoietin induced by anti-angiogenic effect [36]. But the mechanism is not fully understood and further studies are needed.

In 2014, nintedanib (BIBF1120), under the brand name vargatef, was approved in European Union as a second line agent in advanced lung adenocarcinoma [17, 37]. Recent scientific evidence showed that nintedanib in combination with docetaxel prolonged survival of patients with advanced lung adenocarcinoma [37]. Nintedanib is a triple angiokinase inhibitor that simultaneously blocks VEGFR, PDGFR and fibroblast growth factor receptors proangiogenic pathways [38]. These three cellular signal pathways have an important role in angiogenesis, progression and metastasis of malignant tumors [3, 4, 39]. Several novel VEGFR-TKIs are also under clinical investigation. Angiogenesis is imperative for the tumor growth and metastasis. The formation and remodeling of vessels is mediated by stimulating molecules released from malignant cells [4, 40]. The stimulating molecules activate cellular signal pathways resulting in new vessels formation. VEGFR-TKIs inhibit sprouting of the vessels through blocking those activating signal pathways [31, 33]. Unlike classical cytotoxic drugs, VEGFR-TKIs have no direct cell-killing effect on malignant cells. Conventional chemotherapy drugs could directly kill normal and cancerous cells through inhibiting proliferation, interfering metabolism or/and inducing apoptosis. Taken together, the combination of VEGFR-TKIs and chemotherapy results in considerable malignant cells death and rapid tumor shrinkage [32, 37]. One of the advantages is that it shows more effective anti-cancer activity than chemotherapy agent alone. Another advantage is that combination therapy could reduce the dose of cytotoxic drugs, and minimize normal cells death caused by cytotoxic drugs.

Previous studies showed that hypoxia expedited tumor invasion and metastasis by inducing hepatocyte growth factor and hypoxia-inducible factor (HIF) [41, 42]. One interesting question attracts our attention: whether VEGFR-TKIs drive tumor progression and metastasis in hypoxia. The shifting from normoxia (21% O_2_) to hypoxia indeed activates cancer cells for aggressive behavior [41]. However, “normoxia” defined as 21% O_2_ (160 mmHg) is not physiological. The oxygen levels in advanced NSCLC is just about 1% (5 mmHg) [43]. VEGFR-TKIs shift malignant cells from hypoxia (the normal oxygen level in NSCLC) to deeper hypoxia or anoxia. The metastasis and invasion of cancer is stimulated by physiological hypoxia not inflicting hypoxia [44]. VEGFR-TKIs exhaust oxygen and starve cells to death. Thus, metastasis may not occur during VEGFR-TKIs therapy [44, 45]. Effective anti-cancer therapy suppresses proliferation of sensitive cells and only resistant cells survive. The ideal anti-cancer therapy suppresses all malignant cells. There may be no residual cells. Currently, VEGFR-TKIs therapy is limited mainly by low efficacy and shortage of selective drugs. Blagosklonny MV had proposed strategies to increase efficacy: combination of VEGFR-TKIs with metronomic chemotherapy, anti-HIF drugs and inactive prodrugs [44]. Given various potential therapeutic methods, we can imagine effective VEGFR-TKIs therapies of the future.

According to our data, VEGFR-TKIs improved PFS, ORR and DCR in advanced NSCLC. Disappointedly, clinical responses to VEGFR-TKIs therapy did not translate into OS improvements. Another interesting question attracts our attention: why therapeutic responses to VEGFR-TKIs do not prolong the survival of NSCLC patients. Anti-cancer therapies kill proliferating cells and cause remission. Cancer stem cells (CSCs) persist in residual tumor and lead to relapse. All proliferating cells are descendance of the same CSCs. The life expectancy of relapsed tumor is identical to that of initial tumor. So, anti-cancer therapies extend patients' OS time between remission and relapse [46]. However, anti-cancer therapies cannot kill all proliferating cells in NSCLC. Inevitably, CSCs hierarchy shifts to the dominance of proliferating cells [47]. Anti-cancer therapies select proliferating cells with resistance-confirming mutations. Resistant proliferating cells will be attenuated by differentiation, unless proliferating cells acquire the potential for self-renewal [47]. Once the oncogenic resistant mutations render proliferating cells drug-resistant, apoptosis-reluctant and highly malignant, more aggressive relapse tumors may occur. The relapse tumor tends to be more lethal compared to initial tumor. Therefore, there is no improvement of OS. Selection for oncogenic resistance can explain the response-survival paradox. VEGFR-TKIs could improve survival of patients as long as acquired resistance is exploited.

In this meta analysis, the AEs of VEGFR-TKIs were various, because several VEGFR-TKIs are multitargeted tyrosine kinase inhibitors. Cediranib inhibits VEGFR, PDGFR, and c-kit. Sorafenib can inhibit VEGFR and PDGFR tyrosine kinase as well as the Raf kinases. Vandetanib is a potent inhibitor of RET receptor tyrosine kinase, VEGFR and EGFR pathways [40]. The wide clinical use of VEGFR-TKIs has raised concerns over their AEs. Thus, an emerging issue is to identify the predictor for selecting patients who benefit from VEGFR-TKIs. Although several markers have been postulated, such as VEGF, MMP-9 and IL-8 [48], no biomarker has yet been used routinely in NSCLC. So, predictive markers are greatly needed to identify the subset of patients who may gain the utmost benefit from VEGFR-TKIs.

This meta analysis comprehensively analyzed data from different studies to achieve a more robust results. However, several limitations need to be addressed. First, this meta analysis was based on study-level evidence. Thus, confounding factors (demographic characteristics and post-progression treatment) could not be incorporated into analysis. An individual patient data-based meta-analysis would give more reliable results. Second, due to lack of original data, we did not perform sub-analysis based on predictive markers to identify the exact benefit population. Third. our conclusions came from the sum of 23 RCTs; 6 of which were the comparison between case and placebo; 17 of which were the comparison between TKI added on chemotherapy vs chemotherapy alone. The little heterogeneity across studies may enhance the reliability of this study. Although subgroup analyses and meta regression both demonstrated the regimen did not change the overall results significantly, inconsistent HRs and RRs for different regimen should be noticed. Therefore, further research with updated data from individual patient are needed to clarify the efficacy and safety of VEGFR-TKIs.

## MATERIALS AND METHODS

### Search strategy and selection criteria

Two investigators independently searched PubMed, EMBASE, Cochrane Library databases as well as Web of science to identify the articles with the following key words: NSCLC [All Fields]” or “lung cancer [All Fields]” and “cediranib (AZD2171) [All Fields]” or “motesanib (AMG706) [All Fields]” or “nintedanib (BIBF1120) [All Fields]” or “pazopanib (GW786034) [All Fields]” or “sorafenib (BAY43-9006) [All Fields]” or “sunitinib (SU11248) [All Fields]” or “vandetanib (ZD6474) [All Fields]” or “VEGFR [All Fields]” or “TKIs [All Fields]”. Meeting abstracts from the American society of Clinical Oncology, World Congress of Lung Cancer and European Cancer Organization were also hand searched. We also screened the reference lists of review articles and original papers. Results were double-checked and disagreements were resolved by discussion. The published language was limited to English and literature search was conducted up to 5 December 2014.

Results from the initial search that matched the criteria below were eligible. (a) Individuals with advanced NSCLC must be histopathologically confirmed. (b) The studies must be prospective randomized controlled phase II or phase III trials on advanced NSCLC patients. (c) No patient received VEGFR-TKIs or anti-VEGFR antibodies treatment before the randomized controlled trials. (d) The studies must reported one of the four endpoints (PFS, OS, ORR or DCR). For full text review, trials were excluded if (a) clinical trials compared VEGFR-TIKs with anti-VEGF antibodies; (b) data were not available regarding primary or secondary end points; (c) the number of patients for the AEs assessment was not provided; (d) patients with small cell lung cancer or other malignancies or benign lung tumors; (e) patients were not randomized into different groups. If the same patient population was used in more than one study, only the complete study would be included.

### Data extraction and definition

The Preferred Reporting Items for Systematic Reviews and Meta analyses (PRISMA) statements were used to provide complete information about this meta analysis [49]. The 27 items of PRISMA statements were shown in Supplement Checklist 1, which included the title, abstract, methods, results, discussion and funding. According to PRISMA statements, all data were independently extracted by two authors using standardised data compilation forms. The discrepancies were resolved by discussion to validate the accuracy of extraction. The primary end point was defined as PFS to standardize data collection. The secondary end points included OS, ORR, DCR as well as common AEs. The DFS was defined as the time from random assignment to disease progression. The OS time was calculated from random assignment to the date of death from any cause. Tumor response was defined as progressive disease, stable disease, partial response or complete response based on the Response Evaluation Criteria in Solid Tumors criteria [50]. The ORR was defined as the proportion of patients showing complete or partial response. The DCR included stable disease, partial response and complete response for longer than three months. Common Terminology Criteria for Adverse Events (CTCAE 3.0) was used to grade the severity of AEs. The quality of included RCTs was assessed according to Jadad scale [51].

### Statistical analysis

HRs and corresponding 95% CIs were pooled for PFS and OS. The HRs and 95% CIs were extracted as previously reported [52-54]. The most accurate method was to obtain parameters directly from the articles or to calculate the HRs from O-E statistic and variance. The second method was to estimate the HRs from sample size, survival rate at specified times, log rank statistic and P value. Otherwise, Kaplan-Meier Curves were analyzed using the Engauge Digitizer version 4.1 (http://digitizer.sourceforge.net/) to retrieved HRs and 95% CIs. For measurement of RRs and their 95% CIs, we constructed 2×2 tables based on abstracted data from each RCTs. RRs and their 95% CIs were pooled to evaluate overall ORR, DCR and risk of AEs.

The Mantel-Haenszel method was used to determine the choice of the fixed effects model or the random-effects model. A sensitivity analysis was carried out by excluding each study at a time individually. The publication bias was analysed using Begg's funnel plots and Egger's linear regression test [55]. *P* < 0.05 by t-test was defined as significant publication bias. Despite little heterogeneity across studies, meta regression was performed to evaluate potential effects of clinical covariables on overall outcomes. Three categorical variable were investigated, including drug class, treatment line and regimens. Univariate meta-regression analyses were carried out using the random-effects model. The restricted maximum likelihood method was undertaken to estimate the residual between-trial variance and heterogeneity degree [56]. Monte Carlo permutation test was used with 10,000 random permutations [57]. This meta-analysis was carried out using the software Stata 11.0 (Stata Corporation, College Station, TX, USA). All the P values were two-sided. Differences were considered statistically significant at *P* < 0.05.

## CONCLUSIONS

In summary, this study provides proof of principle that VEGFR-TKIs have an advantage in terms of PFS, ORR and DCR, compared with control therapies. However, advanced NSCLC patients treated with VEGFR-TKIs have high risks of AEs. Thus, the monitoring AEs during VEGFR-TKIs therapy is recommended. The risk and benefit of VEGFR-TKIs must be evaluated carefully to select patients who utmost benefit from VEGFR-TKIs treatment.
